# Supplementary material for: What instruments are available to aid or evaluate personalised care delivery, from the perspectives of healthcare practitioners and service users? A narrative scoping review
Source: PLoS One. 2025 Jul 10;20(7):e0325833. doi: 10.1371/journal.pone.0325833 (PMC12244752; doi:10.1371/journal.pone.0325833)
Supplement: S1 File — (DOCX) [file pone.0325833.s001.docx]

| **Search Number** | **Category** | **Search Statement Number** | **Search Terms** |
| --- | --- | --- | --- |
| 1 | Concept – Measure | 1 | measure or evaluation or assessment or outcome or survey or questionnaire or tool or instrument or scale or inventory |
| 2 | Concept – Personalised Care | 2 | personali#ed or care adj/N3 (individual* or plan*, tailor*) or “shared decision” or “support plan*” or self-manag* or “social prescrib*” or activation |
|  | COMBINED | 3 | 1 AND 2 |

Retrieved papers assessed for

1. CONTEXT – health or social care setting.
2. PARTICIPANT – service users/patients OR healthcare staff OR service unit (e.g. team, service, ward, hospital)

**Limiters:**

- English Language
- Adults
- 1990 onwards
