# Supplementary material for: What instruments are available to aid or evaluate personalised care delivery, from the perspectives of healthcare practitioners and service users? A narrative scoping review
Source: PLoS One. 2025 Jul 10;20(7):e0325833. doi: 10.1371/journal.pone.0325833 (PMC12244752; doi:10.1371/journal.pone.0325833)
Supplement: S2 File — (DOCX) [file pone.0325833.s002.docx]

| Authors | Name of Instrument | Short Name | Type of Tool | Items (n) | Response Categories | Factors (n) | Domain Descriptors | Population | Clinical Group and Sub-Group | Setting | Country | Area of Personalised Care |
| --- | --- | --- | --- | --- | --- | --- | --- | --- | --- | --- | --- | --- |
| Allen and Jones (1998) | Asthma General Knowledge Questionnaire | AGKQA | Questionnaire | 31 | True, False, Not Sure | 4 | Asthma knowledge: aetiology, pathophysiology; medications; severity; and symptom management including trigger minimisation and exercise. | Patients | Respiratory; Asthma | Primary Care - Outpatient | Australia | Supported Self-Management |
| Amano (2023) | Self-Management Scale - Lower Urinary Tract Symptoms - Radical Prostatectomy | SMS-LUTS-RP | Questionnaire | 18 | 6-point scale from 0 = "not at all applicable" to 5 = "extremely applicable" | 5 | Monitoring urinary status; coping with life difficulties due to LUTS; collaboration with medical professionals; continued training to improve LUTS; living with LUTS. | Patients | Cancer; Radical Prostatectomy | Primary Care - Clinic | Japan | Supported Self-Management |
| Mbuagbaw et al (2017); Aronson et al (2018) | Skills, Confidence and Preparedness Index | SCPI | Questionnaire | 25 | 10 point scale from 1 = "very little" to 10 - "a lot" | 3 | Skills, Confidence and Preparedness | Patients | Endocrine; T1 and T2 Diabetes | Primary Care - Clinic | Canada | Supported Self-Management |
| Arvanitis et al (2020) | Influence and Motivation for Patient Activation in Diabetes Care | IMPACT-D^TM^ | Questionnaire | 6 | 5 point scale: never, rarely, sometimes, often, always (for values) and not at all, a little bit, somewhat, quite a bit, very much (for influence) | 2 | Values (4 items); influence (2 items) | Patients | Endocrine; Type 2 Diabetes | Primary Care - Clinic | USA | Supported Self-Management |
| Elwyn et al (2013); Barr et al (2014) | CollaboRATE | CollaboRATE | Questionnaire | 3 | 5 point scale: "no effort was made" to "every effort was made" | NA | NA | Patients | Mixed LTC | Healthy Population + Primary Care | USA | Shared Decision Making |
| Elwyn et al (2013) | OPTION5 | OPTION5 | Observational Scale | 5 | 0-4 where 0 represents absence of a Shared Decision Making specific competency and 4 represents optimal performance. Max score = 20; re-scaled to a score out of 100. | 1 | Shared Decision Making | Patients | Mixed LTC | Primary Care - GP | USA | Shared Decision Making |
| Elwyn et al (2003) | Observing Patient Involvement in Decision Making | OPTION | Observational Scale | 12 | 0-4 magnitude scale - from "the behaviour is not observed" to "the behaviour is observed and executed to a high standard" | NA | NA | Clinicians | Healthcare Professionals; GP | Primary Gare | UK | Shared Decision Making |
| Bartlett and Peterson (2013) | Shared Decision Making Inventory - Revised | Shared Decision Making-R | Questionnaire | 18 | 5 item response rate – not at all confident to extremely confident; strongly agree to strongly disagree | 4 | Knowledge, attitudes, self-efficacy and intent | Clinicians | Healthcare Professionals; School Nurse - Vaccination | NA | USA | Shared Decision Making |
| Bayliss et al (2005) | No title given |  | Questionnaire |  | 1-5 from "not at all" to "a lot" [how much each item interfered with daily activities] | 13 | General barriers to self-care; low level of physical functioning; inappropriate social support; lack of patient-provider communication; symptoms of depression; financial constraints; knowledge about conditions; care recommendations for different conditions; low self efficacy; inconvenience of obtaining care; overwhelming effect of one condition | Patients | Mixed LTC; Multimorbidity | Inpatient and Outpatient | USA | Supported Self-Management |
| Bennett et al (2017) | Cochlear Implant Management Skills Survey | CIMS-self | Questionnaire | 11 | 4 point scale; self-rating ability to perform each task graded as ‘Never/Unsure’, ‘Sometimes’, ‘Most of the time’ and ‘Always’ |  | Not described | Patients | Audiology; Cochlear Implant | Clinic | Australia | Supported Self-Management |
| Bishop and Frain (2011); Ghahari et al (2014) | Multiple Sclerosis Self-Management Scale | MS Supported Self-Management | Questionnaire | 24 | 5-point scale (1 = completely disagree, 2 = somewhat disagree, 3 = neither agree nor disagree, 4 = somewhat agree, 5 = completely agree) | 5 | Health Care Provider Relationships/Communication; Treatment Adherence/Barriers; Socia/Family Support; MS Knowledge and Info; Health Maintenance Behaviour | Patients | Neurology; Multiple Sclerosis | Community | USA/ Canada | Supported Self-Management |
| Boger et al (2015) | Southampton Stroke Self-Management Questionnaire | Supported Self-ManagementQ | Questionnaire | 28 | 6 point style (Always true = 6, Always false = 1) | 3 | Attitudes, Skills, Behaviours | Patients | Neurology; Stroke | Community | UK | Supported Self-Management |
| Bomhof-Roordink et al (2020) | iSHARE Patient | na | Questionnaire | 15 | 6 point scale from "not at all" to "completely" | 6 | Choice awareness, medical information, preferences, deliberation, time for deliberation, decision | Patients | Cancer | Clinic | Netherlands | Shared Decision Making |
| Bomhof-Roordink et al (2020) | iSHARE Physician | na | Questionnaire | 15 | 6 point scale from "not at all" to "completely" | 6 |  | Clinicians | Cancer | Clinic | Netherlands | Shared Decision Making |
| Bond et al (2020) | Head and Neck Cancer Patient Self-Management Inventory | HNC - PSMI | Questionnaire | 24 | Whether or not the patient performs the task (Yes/No) and difficulty performing the task (5-point scale; 1 = not at all difficult, 5 = extremely difficult) | 11 | General function; medical care; nutrition; tube feeding; tracheostomy care; oral care; speech and voice; skin care; rehabilitation; psychosocial support; symptom management | Patients | Cancer; Head and Neck Cancer | Community | USA | Supported Self-Management |
| Caihong et al (2013) | COPD Self-Management Scale | CSMS | Questionnaire | 51 | 5-point scale (1=never, 2=rarely, 3=sometimes, 4=often, and 5=always) | 5 | Symptom Management; Daily Life Management; Emotion Management; Information Management; Self Efficacy | Patients | Respiratory; COPD | Inpatient and Outpatient | China | Supported Self-Management |
| Carlton et al (2017) | Health and Self-Management in Diabetes | HASMID^V1^ | Questionnaire | 8 | 4 response levels, tailored to each question - never, sometimes, usually, always. | 2 | Quality of Life, Self-Management | Patients | Endocrine; Diabetes | Community | UK | Supported Self-Management |
| Carlton et al (2020) | Health and Self-Management in Diabetes - 10 | HASMID-10 | Questionnaire | 10 | 4 response levels, tailored to each question - never, sometimes, usually, always. | 2 | Quality of Life, Self-Management | Patients | Endocrine; Diabetes | Community | UK | Supported Self-Management |
| Kerns et al (1997) | Pain Stages of Change Questionnaire | PSOCQ | Questionnaire | 49 | five-point scale, from strongly disagree (1) to strongly agree (5) | 4 | precontemplation, contemplation, action, and maintenance | Patients | Pain Chronic Pain | Tertiary Care | USA | Supported Self-Management |
| Chen et al (2023) | Self-Management Behaviours Scale in Rheumatoid Arthritis | RA-SMBS | Questionnaire | 23 | Always, Often, Occasionally, Sometimes, Never | 4 | Medication management, resource utilization, emotional management and symptom management | Patients | MSK Rheumatoid Arthritis | Tertiary Care | China | Supported Self-Management |
| Chung et al (2023) | Kidney Transplant Self-Management Scale | KT-SMS | Questionnaire | 16 | 5-point scale with 1 = *strongly disagree*, 2 = *disagree*, 3 = *neutral*, 4 = *agree*, and 5 = *strongly agree* | 5 | Medication adherence, cardiovascular risk reduction, protecting kidney, ownership, skin cancer prevention | Patients | Renal Kidney Transplant |  | USA | Supported Self-Management |
| Coulombe et al (2015) | Mental Health Self-Management Questionnaire | MHSMQ | Questionnaire | 18 | Five-point response scale: “Never” (0), “Very rarely” (1), “Rarely” (2), “Often” (3), and “Very often” (4). | 3 | Clinical, Empowerment and Vitality | Patients | Mental Health Anxiety and Depression | Primary Care | Canada | Supported Self-Management |
| Schuurman et al (2005) | Self-Management Scale 30 | SMAS-30 | Questionnaire | 30 | 6 point scale - wording dependent on domain | 6 | Taking Initiatives; Investment Behaviour; Self Efficacy; Variety; Multifunctionality; Positive Frame of Mind | Patients | Geriatrics Slight to moderate frailty | Primary Care [homes for the elderly, sheltered living and recreational clubs] | Netherlands | Supported Self-Management |
| Cramm et al (2012) | Self-Management Scale - Short Version | SMAS-S | Questionnaire | 18 | 6 point scale - wording dependent on domain | 6 | Taking Initiatives; Investment Behaviour; Self Efficacy; Variety; Multifunctionality; Positive Frame of Mind | Patients | Geriatrics Post Hospital Admission 65+ | Primary Care | Netherlands | Supported Self-Management |
| Dai et al (2023) | Diabetic Self-Management Scale | DSMS | Questionnaire | 22 |  | 3 | Information; Motivation; Behaviour Skills | Patients | Endocrine T2 diabetes | Primary Care | China | Supported Self-Management |
| Day et al (1996) | The Ipswich Diabetes Self-Management Questionnaire | IDSMQ | Questionnaire | 35 | 5 point scale | 9 | Effect on lifestyle; self efficacy; weight concern; perceived goals; cost-benefits; other people; diet barrier; emotional adjustment | Patients | Endocrine; Diabetes | Primary Care | UK | Supported Self-Management |
| Devia et al (2022) | CAPABLE | CAPABLE | Observational assessment | 16 | NA - each item is observed by the clinician, to see if the person can perform it independently (Y/N). | 3 | Cognitive, Sensory, Motor | Patients | Renal; Peritoneal Dialysis | Primary Care | Brazil | Supported Self-Management |
| Eikelenboom et al (2015) | Self-Management Screening Tool | SeMaS | Questionnaire | 27 | Range of 4, 5 and 10 point scales | 8 | Self efficacy, locus of control, depression, anxiety, coping, social support, perceived burden of disease, type of support [computer skills, functioning in groups, willingness to perform self monitoring] | Patients | Mixed LTC Diabetes, Asthma, COPD, CVD | Primary Care | Netherlands | Supported Self-Management |
| Escoffery et al (2015) | Adult Epilepsy Self-Management Measurement Instrument | AESMMI |  | 65 | 5-point scale (i.e., 1 = none to 5 = all of the time, 1 = never to 5 = always, 1 = not at all to 5 = completely true) or “Not applicable” (i.e., missing value). | 11 | Health-care Communication, Coping, Treatment Management, Seizure Tracking, Social Support, Seizure Response, Wellness, Medication Adherence, Safety, Stress Management, and Proactivity | Patients | Neurology; Epilepsy | Primary Care | USA | Supported Self-Management |
| Eton et al (2017) | Patient Experience with Treatment and Self-Management | PETS | Questionnaire | 48 | 5 point response scale; wording dependent on domain | 9 | Medical information, medications, medical appointments, monitoring health, interpersonal challenges, medical/healthcare expenses, difficulty with healthcare services, role/social activity limitations, and physical/mental exhaustion | Patients | Mixed LTC; Multimorbidity | Primary Care | USA | Supported Self-Management |
| Gifford at al (2022) | Family Caregiver Activation in Transitions | FCAT | Questionnaire | 6 | 5 point scale | 1 | Activation | Carers | Hospital Discharge | Transitional Care [from ED] | USA | Supported Self-Management |
| Glasgow et al (2000) | Chronic Ilness Resources Survey | CIRS | Questionnaire | 64 or 29 | 5 point scale + perceived importance |  | Personal, Family and friends, physician and health team; neighbourhood/community, Organisations, Worksites, Media and Policy | Patients | Mixed LTC | Primary Care | USA | General |
| Glasgow et al (2004) | Diabetes Problem-Solving Inventory | DPSI | Questionnaire | 9 | Open text responses - participants given a hypothetical scenario then asked how they would deal with it. Responses then scored by a "coder" - using 5 point scale [1 = very poor strategy to 5 = excellent strategy] | 3 | Healthful Easting; Physical Activity; Stress Management | Patients | Endocrine Post Menopausal; diabetes | Primary Care | USA | Supported Self-Management |
| Valentine et al (2021) | Shared Decision Making Process Scale | Shared Decision Making Process Scale |  | 4 | 2 or 3 point binary response - Yes/No or Some/A Little/Not at all | 4 | Options, pros, cons and preferences | Patients | Surgery | Secondary Care | USA | Shared Decision Making |
| Greene et al (2017) | Clinician Self-Management Support Strategies Measure | SMS | Questionnaire | 9 | 5 point scale - never to very often. | 5 | Emphasizing patient ownership; partnering with patients; identifying small steps for change; having frequent follow up; showing patients care and concern | Clinicians | Healthcare Professionals; Primary Care Providers | Primary Care | USA | Supported Self-Management |
| HyunSoo et al (2018) | Arthritis Self-Management Assessment Tool | ASMAT | Questionnaire | 32 | 4 point scale; never to always | 3 | Medical, Behavioural, Psycho-emotional | Patients | MSK; Arthritis | Primary Care | South Korea | Supported Self-Management |
| Karahan Okuroglu et al (2020) | Insulin Treatment Self-Management Scale | IT-SMS | Questionnaire | 32 | 5 point scale | 3 | Behavioural, Cognitive, Affective | Patients | Endocrine Diabetes; insulin management | Secondary Care | Turkey | Supported Self-Management |
| Kephart et al (2022) | Patient Reported Inventory of Self-Management of Chronic Conditions | PRISM-CC | Questionnaire | 36 | 6 point response scale, depending on question [difficulty; level of agreement; frequency] | 6 | Resource, Process, Internal, Activity, Social Interaction, Healthy Behaviour, Disease Control, | Patients | Mixed LTC; Chronic Disease | Primary Care | Canada | Supported Self-Management |
| Kolbe et al (1996) |  |  |  | 2 | Open discussion | NA | NA | Patients | Respiratory; Asthma | Primary Care | New Zealand | Supported Self-Management |
| Kong et al (2018) | Chronic Hepatitis B Self-Management Scale | CHBSMS | Questionnaire | 25 | 5 point - never to always | 4 | Symptom Management; Lifestyle Management; Psychosocial Coping; Disease Information Management | Patients | Haematology; Hepatitis B | Primary Care | China | Supported Self-Management |
| Kosmala-Anderson et al (2011) | Practices in Self-Management Support | PSMS | Questionnaire | 25 |  | 3 | Clinical Self-Management Support, Patient Centeredness, Organisational Self-Management Support | Clinicians | Healthcare Professionals; Clinicians working with people with LTC | Primary Care | UK | Supported Self-Management |
| Simon et al (2006) | Shared Decision Making Questionnaire | Shared Decision Making-Q-9 | Questionnaire | 11 | 4 point Likert scale | 1 | Shared Decision Making | Patients | Mixed LTC; Depression; gynaecology; urology; anaesthesia; general practice | Primary and Secondary Care | Germany | Shared Decision Making |
| Kriston et al (2010) | Shared Decision Making Questionnaire - 9 Item | Shared Decision Making-Q-9 | Questionnaire | 9 | 6 point scale - completely disagree to completely agree | 1 | Shared decision making | Patients | Mixed LTC; MSK, CVD, Endocrine, GI, Infection, Neuro | Primary Care | Germany | Shared Decision Making |
| Kuang et al (2022) | Self- management Scale for Chinese pregnant woman with GDM | NA | Questionnaire | 35 | 5 point agreement scale | 4 | Self-Management consciousness; pregnancy management; blood glucose management; resource utilization | Patients | Pregnancy | Primary Care | China | Supported Self-Management |
| Lee et al (2020) | Diabetes Self-Management Scale | DSMS | Questionnaire | 17 | 5 point Scale | 6 | Self Regulation; Blood Glucose Monitoring; Physical Exercise; Taking Medication; Diet; Stress Alleviation | Patients | Endocrine; Type 2 Diabetes | Secondary Care | South Korea | Supported Self-Management |
| Lin et al (2008) | Diabetes Self-Management Instrument | DSMI | Questionnaire | 33 |  | 5 | Self integration; self regulation; interaction with health professionals and significant others; self-monitoring of blood glucose; adherence to recommended regimen | Patients | Endocrine; Type 2 Diabetes | Secondary Care | Taiwan | Supported Self-Management |
| Lin et al (2013) | Chronic Kidney Disease Self-Management Instrument | CKD-SM | Questionnaire | 29 |  | 4 | Self integration; problem solving; seeking social support; adherence to recommended regimens | Patients | Renal; CKD | Secondary Care | Taiwan | Supported Self-Management |
| Lo et al (2022) | Stroke Self-Management Behaviours Performance Scale | SSBPS | Questionnaire | 11 | 11 point rating scale from 0 (very dissatisfied) to 10 (very satisfied) | 2 | Medical Management; Psychosocial Management | Patients | Neurology; Stroke | Secondary Care | Hong Kong | Supported Self-Management |
| Ludman et al (2002) | Health Care Climate Questionnaire | HCCQ | Questionnaire | 10 | 7 item - strongly agree to strongly disagree | 1 | Perceptions of provider support | Patients | Mental Health; Bipolar Disorder | Primary Care | USA | Supported Self-Management |
| Mancuso et al (2009) | Asthma Self-Management Questionnaire | ASMQ | Questionnaire | 16 | Multiple choice - 5 possible responses per item | 3 | Preventative Strategies, Inhaler Use, Medications [knowledge of] | Patients | Respiratory; Asthma | Primary Care | USA | Supported Self-Management |
| Manhas et al (2020) | Alberta Shared Decision Making Instrument | ASK-MI | Questionnaire | 6 | 6 point scale + "not applicable" option | 1 | Shared Decision Making | Patients | Rehabilitation | Primary Care | Canada | Shared Decision Making |
| Mares et al (2022) | Heart Health Self-Efficacy and Self-Management Scale | HH-SESM | Questionnaire | 12 | 4 point scale: Not confident to very confident (1-4) and rarely/never to always (1-4) | 2 | Self efficacy and self-management | Patients | Cardiac; CVD | Secondary Care | Australia | Supported Self-Management |
| McCabe et al (2020) | Knowledge about Atrial Fibrillation and Self-Management Survey | KAFSM | Questionnaire | 28 | Multiple choice format - correct response, two distractor responses, and I don’t know. | 8 | What Is AF; common symptoms; consequences; recurrent nature; treatments; monitoring; risk factors psychological response | Patients | Cardiac; AF | Primary Care | USA | Supported Self-Management |
| McCaskill et al (2016) | Self-Care Utility Geriatric African-American Rating | SUGAAR | Questionnaire | 25 | Dichotomous - yes or no | 1 | Diabetes self-management | Patients | Endocrine; Diabetes T2 | Primary Care | USA | Supported Self-Management |
| Mei-Hua et al (2017) | Scale for Evaluating Self-Management Needs of Knee Osteoarthritis | SMNKOA | Questionnaire | 35 | 5 point - extremely not needed to extremely needed | 3 | Monitoring and solving symptoms; seeking community resources; life adjustments and amendments. | Patients | MSK; Knee Osteoarthritis | Secondary Care - Clinics | Taiwan | Supported Self-Management |
| Michon et al (2011) | Illness Self-Management for Psychiatric Vocational Rehabilitation | ISM-PVR | Questionnaire | 14 | NA | 3 | Vocational goal; perceived illness related barriers to goal achievement; self-management strategies | Patients | Mental Health | Secondary Care - Vocational Rehab | Netherlands | Supported Self-Management |
| Nakawatase et al (2007) | Evaluation Scale for Self-Management Behaviour related to Physical Activity in Type 2 Diabetes | ES-SMBPA-2D | Questionnaire |  |  | 2 | Self-Management Behaviour a) enhance physical activity and b) maintain physical activity | Patients | Endocrine; Diabetes T2 | Primary Care | Japan | Supported Self-Management |
| Obad et al (2023) | No reported name |  | Questionnaire | 56 |  | 8 |  | Patients | Endocrine; Diabetes | Primary Care | USA | Supported Self-Management |
| Osborne et al (2007) | Health Education Impact Questionnaire | heiQ | Questionnaire | 42 | 6 point scale - strongly disagree to strongly agree [later refined to have 40 items and a 4 point response scale] | 8 | Positive and active engagement in life; heath directed behaviour; skill and technique acquisition; constructive attitudes and approaches; self monitoring and insight; health service navigation; social integration and support; emotional wellbeing | Patients | Mixed LTC | Primary Care | Australia | Supported Self-Management |
| Paxton et al (2015) | Sedentary Behaviour Strategy Sel-Management Scale | SBSMS | Questionnaire | 15 | 5 point scale from never to many times | 1 | NA | Patients; African-American | Cancer; Breast Cancer | Secondary Care | USA | Supported Self-Management |
| Peng and Wu (2020) | Oral Chemotherapy Self-Management Scale | OCSMS | Questionnaire | 36 | 4 point ordinal rating scale - not relevant to very relevant | 5 | Daily life management; symptom management; medication management; emotional cognitive management; social support | Patients | Cancer; Chemotherapy | Secondary Care | China | Supported Self-Management |
| Planalp et al (2022) | Barriers and Supports Evaluation | BASES | Questionnaire | 30 |  | 5 | Learning opportunities; costs and insurance; family and friends; coping and behavioural skills; diabetes provider interactions | Patients | Endocrine Type 1 Diabetes | Primary Care | USA | Supported Self-Management |
| Riegel et al (2000) | Self-Management of Heart Failure |  | Questionnaire | 6 | 3 or 4 point response options - importance, ease, confidence etc, depending on question. | 6 | Recognizing a change; evaluating the change; implementing a treatment strategy; evaluating the treatment strategy; ease of evaluating the treatment strategy; self-efficacy | Patients | Cardiac Heart Failure | Primary Care | USA | Supported Self-Management |
| Schmitt et al (2013) | Diabetes Self-Management Questionnaire | DSMQ | Questionnaire | 16 | 0-3, applies to be very much to does not apply to me | 4 | Glucose Management; Dietary Control; Physical Activity; Health Care Use | Patients | Endocrine Diabetes Type 1 and 2 | Primary Care | Germany | Supported Self-Management |
| Scholl et al (2012) | Shared Decision Making Questionnaire 9 - Physician | Shared Decision Making-9-Doc | Questionnaire | 9 | 6 point scale, completely disagree to completely agree | 1 | Shared Decision Making process | Clinicians | Healthcare Professionals Physicians | Primary Care | Germany | Shared Decision Making |
| Smalley et al (2022) | Self-Management Abilities Test | SMAT | Questionnaire | 20 | Specific knowledge questions with multiple response choices; one of which is correct. | 5 | General health knowledge; bronchiectasis specific knowledge; symptom management; communication, addressing deterioration | Patients | Respiratory; Bronchiectasis | Primary Care | UK | Supported Self-Management |
| Thorpe et al (2007) | Vasculitis Self-Management Scale | VSMS | Questionnaire | 43 | 5 point scale | 8 | Medication; Health services; Infection; Diet; Exercise; Symptom Monitoring; Reporting; Adjusting activities | Patients | Vascular; Vasculitis | Primary Care | USA | Supported Self-Management |
| Tokunaga-Nakawatase et al (2012) | Evaluation scale for self-management behaviour related to physical activity of patients with coronary heart disease | ES-SMBPA-CHD | Questionnaire | 39 | 5-point scale, never to always | 2 | Self-management behaviour to enhance physical activity in daily life; behaviour to maintain the level of activity | Patients | Cardiac; Coronary Heart Disease | Primary Care (Outpatient) | Japan | Supported Self-Management |
| Shelton Smith et al (1995) | The Perceived Health Competence Scale | PHCS | Questionnaire | 8 | 5 point strongly agree to strongly disagree | 1 | Capability in effectively managing own health outcomes | Patients | MSK; Rheumatoid Arthritis | Secondary Care and General Public | USA | Supported Self-Management |
| Wang et al (2023) | Character Strengths Use in Diabetes Self-management Scale |  | Questionnaire | 12 |  | 3 | Learning proactivity, taking on challenges; thinking positively | Patients | Endocrine; Type 2 diabetes |  | Taiwan | Supported Self-Management |
| Wang et al (2015) | Self-Management Scale for Peritoneal Dialysis |  | Questionnaire | 28 | 4 point scale - Never, Sometimes, Often, Always | 5 | Solution bag replacement; trouble shooting during operation; diet management; complication monitoring; emotion management; return to social life | Patients | Renal; Peritoneal Dialysis | Secondary Care - Inpatients | China | Supported Self-Management |
| Webel et al (2012) | HIV Self-Management Scale |  | Questionnaire/Scale | 20 | 0 indicated the item was not applicable to the individual participant, 1 meant the individual item occurred none of time, 2 meant the item occurred some of the time, and 3 indicated the item occurred all the time for the individual participant. | 3 | Daily self-management health practices; social support and HIV management; chronicity of HIV self-management | Patients | Infectious Diseases; HIV and AIDS | Primary Care | USA | Supported Self-Management |
| Wegener et al (2014) | Readiness to Engage in Self-Management after Acute Traumatic Injury | RESMATI | Questionnaire |  | Not reported | 3 | Pre-contemplation; contemplation; action/maintenance | Patients | Major Trauma | Primary Care | USA | Supported Self-Management |
| Wehmeier et al (2020) | The Self-Management Self-Test | SMST | Questionnaire | 5 | 5 point scale - very badly to very well | 5 | Awareness, Relationships, Planning, Decision Making, Action | Patients | Mental Health; Major Depression | Secondary Care - Inpatients | Germany | Supported Self-Management |
| Wu et al (2023) | The Self-Management Capability, Support and Motivation-Behaviour Scale |  | Questionnaire | 33 | 5 point scale - strongly agree to strongly disagree or very consistent to vert inconsistent (dependent on domain) | 4 | Capability, Support, Motivation, Behaviour | Patients | Geriatrics; Hypertension | Secondary Care | China | Supported Self-Management |
| Yun et al (2015) | Smart Management Strategy for Health Assessment Tool | SAT | Questionnaire | 91 | 4 point Likert Never, Sometimes, Always, Never | 16 | Proactive problem solving; positive reframing creating empowered relationships; experience sharing; goal and action setting; rational decision making; healthy environment; priority based planning; life value pursuing; self sustaining; self-motivating; activity coping; self implementing (maintaining); reflecting, energy conserving. | Patients | Cancer | Secondary Care - Inpatients | Korea | Supported Self-Management |
| Zhu et al (2020) | Self-Management of Type 1 Diabetes for Chinese Adults (SMOD-CA) scale | SMOD-CA | Questionnaire | 30 | 5 point scale - never to always | 4 | Daily performance of self-management; disease management and collaboration; coping with disease related problems; goals of self-management | Patients | Endocrine ; Type 1 Diabetes | Primary Care | China | Supported Self-Management |
| Oh et al (2023) | Knowledge: Cardiac Disease and Self-Management: Cardiac Disease |  | Assessment Framework | 60 | 5 point - never demonstrated to consistently demonstrated | 2 | Knowledge, Self-Management | Patients | Cardiac; CVD |  | USA | Supported Self-Management |
| Degner et al (1997) | Control Preferences Scale | CPS | Assessment - 5 cards portraying different roles consumers could assume in treatment decision making. | 5 | Analysed according to order of card placement | 1 | Control preferences | Patients | Mixed LTC |  | USA | Shared Decision Making |
| O'Connor (1995) | Decisional Conflict Scale | DCS | Questionnaire |  |  | 3 | Uncertainty; Effective Decision Making; Factors Contributing to Uncertainty | Patients | All - tested in influenza vaccination and breast cancer screening | Primary Care | Canada | Shared Decision Making |
| Kim et al (2001) | Kim Alliance Scale | KAS | Questionnaire | 30 | Behavioural 4 point Likert from 1 (never) to 4 (always) | 4 | Collaboration, Communication, Integration, Empowerment | Patients | General Health | General Public | USA | General |
| Kim et al (1991) | Osteoporosis Health Belief Scale |  |  | 35 | 5 point scale | 6 | Susceptibility; Seriousness; Health Motivation; Barriers; Benefits related to calcium; Benefits related to exercise | Patients | MSK; Osteoporosis | Community | USA | Supported Self-Management |
| [Battersby et al](https://doi.org/10.1071/PY03022)  (2003) | Partners in Health Scale | PIH | Questionnaire | 11 | 9 point rating scale - 0’ indicated good self-management and ‘8’ poor Self-Management | 3 | Core self-management; condition knowledge; symptom monitoring | Patients | Mixed LTC; Range of different chronic conditions | Primary Care - GP | Australia | Shared Decision Making |
| Lerman et al (1990) | Patients Perceived Involvement in Care Scale | PICS | Questionnaire | 13 |  | 3 | Doctor Facilitation; Patient-Physician Information Exchange; Patient Decision Making | Patients | Mixed LTC | Primary Care | USA | General |
| Bennett et al (2009) | Preparation for Decision Making Scale | PrepDM | Questionnaire | 10 | 5 - not at all to a great deal | 1 |  | Patients | MSK; Orthopaedics |  | USA | Shared Decision Making |
| Toobert et al (2000) | Summary of Diabetes Self-Care Activities | SDSCA | Questionnaire | 11 or 25 | Vary depending on question - but mostly score "in how many of the past 7 days have you….." | 6 | General Diet; Specific Diet; Exercise; Blood Glucose Testing; Foot Care; Smoking | Patients | Endocrine; Diabetes Type 2 | Primary Care | USA | Supported Self-Management |
| Joby et al (2024) | Expanded Catheter Self-Management Scale | E-CSM | Questionnaire | 23 | 3 category ordinal scale (1 = not at all, 2 = sometimes, and 3 = most or all of the time). | 5 | Self-monitoring of catheter; proactive, help-seeking behaviour; bowel self-care; hygeine-related  d catheter site; drainage bag care. | Patients | Renal; in-dwelling urinary catheter | Community | Australia | Supported Self-Management |
| Beauvais et al (2023) | The Spondylo -arthritis Knowledge Questionnaire | SPAKE | Questionnaire | 42 | True, False, I don't know | 6 | Knowledge of disease, comorbidities, pharmacological treatments; nonpharmacological treatments; self-care; adaptive skills | Patients | Rheumatology; spondyloarthritis | Community | France | Supported Self-Management |
| Bloom et al (2024) | Patient Cirrhosis Knowledge Questionnaire | NA | Questionnaire | 10 | Multiple Choice - 4 responses options | 1 | Knowledge | Patients | Gastroenterology; liver cirrhosis (2 versions - compensated and decompensated cirrhosis) | Community | USA | Supported Self-Management |
| Dai et al (2023) | Diabetes Self-Management Survey | DSMS | Questionnaire | 22 | Dichotomous - Yes; No for information section; 5 point scale for motivation section; 3 point response - Yes; No; Unsure for behavioural skill section. | 3 | Information; Motivation; Behaviour Skills | Patients | Endocrine; T2 diabetes | Community | China | Supported Self-Management |
| Davis et al (2024) | Patient Assessment of Care in Chronic Conditions | PACIC+ | Questionnaire | 26 | 5 point scale from Never to Almost Always | 6 | Patient activation; design system/design support; goal setting; problem solving; follow up/coordination; 5A's (Ask, Advise, Assess, Assist, Arrange) | Patients | Mixed LTC | Community | Australia | Supported Self-Management |
| Iwaya and Sato (2024) | COPD Self-Care Assessment Scale | CSCS | Questionnaire | 14 | 5 point scale from always to never | 7 | Psychological management; smoke measures; decision making; infection prevention; symptom management; inhaler management; health management for medicine | Patients | Respiratory; COPD | Community | Japan | Supported Self-Management |
| Llahana and Yuen (2024) | Treatment Adherence, Satisfaction and Knowledge Questionnaire. | TASK-Q | Questionnaire | 22 | 5 point scale, depending on question (never to always; strongly agree to strongly disagree) | 2 | Satisfaction and Knowledge; Adherence | Patients | Endocrine; hypothalamic pituitary disorders | Community | UK | Supported Self-Management |
| Vallis et at (2024) | Impact of Glucose Monitoring on Self-Management Scale | ICMSS | Questionnaire | 22 | 5 point scale strongly disagree to strongly agree | 3 | Capability, Opportunity, Motivation | Patients | Endocrine; diabetes | Community | Canada | Supported Self-Management |
| Wu et al (2023) | Self-Management Capability, Support and Motivation-Behaviour Scale |  | Questionnaire | 33 | 5 point scale - strongly disagree to strongly agree; or very inconsistent to very consistent | 4 | Capability, Support, Motivation, Behaviour | Patients | Cardiac; hypertension in elderly people | Community | China | Supported Self-Management |
| Yang et al (2024) | Home-Based Cardiac Rehabilitation Self-Management Scale |  | Questionnaire | 21 | 5 point scale from very inconsistent to very consistent | 5 | Medication management; exercise management; nutrition management; psychological management; risk factor management | Patients | Cardiac; rehabilitation | Community | China | Supported Self-Management |
| de Haas et al (2024) | Mental Health Self-Direction Scale | MHSD | Questionnaire | 19 | 1-6 (does not apply at all to fully applicable) | 4 | Understanding, Demoralization, Commitment, Actorship | Patients | Mental Health | Outpatient | Netherlands | Supported Self-Management |
| Jaarsma et al (2003) | European Heart Failure Self-Care Behaviour Scale | EHFScBS | Questionnaire | 12 | 5 point scale from I completely agree to I completely disagree | 3 | Complying with regimen; asking for help; adapting activities | Patients | Cardiac; heart failure | Community | Netherlands | Supported Self-Management |
| Glasgow et al (2004) | Patient Assessment of Chronic Illness Care | PACIC | Questionnaire | 20 | 5 point scale fron no/never to yes/always | 5 | Patient activation, Delivery System Design/Decisional Support, Problem Solving/Contextual Counselling, Follow-up/Coordination | Patients | Mixed LTC | Community | USA | Supported Self-Management |
| Giguère et al (2020) | I can Shared Decision Making | IcanSDM | Questionnaire | 8 | 10 points scale from strongly agree to strongly disagree | 1 | Perceptions of SDM | Clinicians | Healthcare Professionals | Primary Care | Canada | Supported Self-Management |
| Berkowtz et al (2021) | incorpoRATE |  | Questionnaire | 7 | 0-100 variable analogue scale | 7 | necessity in practice; patient desirability; effective resource use; confidence in skill; importance despite clinical preference; external evaluation; comfort with incongruent patient choice. | Clinicians | Doctors | Primary Care | USA | Supported Self-Management |
